# Does the miR-105–1-Kisspeptin Axis Promote Ovarian Cell Functions?

**DOI:** 10.1007/s43032-024-01554-3

**Published:** 2024-04-17

**Authors:** Zuzana Fabová, Barbora Loncová, Abdel Halim Harrath, Alexander V. Sirotkin

**Affiliations:** 1 Faculty of Natural Sciences, Constantine the Philosopher University in Nitra, Nitra, Slovakia; 2 Department of Zoology, College of Science, King Saud University, 11451 Riyadh, Saudi Arabia; 3 Department of Zoology and Anthropology, Constantine the Philosopher University, Tr. A. Hlinku 1, 949 74 Nitra, Slovakia

**Keywords:** miR-105–1, Kisspeptin, Porcine ovary, Hormones, Apoptosis, Proliferation

## Abstract

The objective of this study was to elucidate the intricate interplay among miR-105–1, kisspeptin, and their synergistic influence on basic ovarian granulosa cell functions. The effects of miR-105–1 mimics or miR-105–1 inhibitor, kisspeptin (0, 1, and 10 ng/ml), and its combinations with miR-105–1 mimics on porcine granulosa cells were assessed. The expression levels of miR-105–1, viability, proliferation (accumulation of PCNA, cyclin B1, XTT-, and BrdU-positive cells), apoptosis (accumulation of bcl-2, bax, caspase 3, p53, TUNEL-positive cells), proportion of kisspeptin-positive cells, and the release of steroid hormones and IGF-I were analyzed. Transfection of cells with miR-105–1 mimics promoted cell viability and proliferation, the occurrence of kisspeptin, and the release of progesterone and IGF-I; in contrast, miR-105–1 mimics inhibited apoptosis and estradiol output. MiR-105–1 inhibitor had the opposite effect. Kisspeptin amplified the expression of miR-105–1, cell viability, proliferation, steroid hormones, and IGF‐I release and reduced apoptosis. Furthermore, the collaborative action of miR-105–1 mimics and kisspeptin revealed a synergistic relationship wherein miR-105–1 mimics predominantly supported the actions of kisspeptin, while kisspeptin exhibited a dual role in modulating the effects of miR-105–1 mimics. These findings not only affirm the pivotal role of kisspeptin in regulating basic ovarian cell functions but also represent the inaugural evidence underscoring the significance of miR-105–1 in this regulatory framework. Additionally, our results show the ability of kisspeptin to promote miR-105–1 expression and the ability of miR‐105–1 to promote the occurrence and effects of kisspeptin and, therefore, indicate the existence of the self‐stimulating kisspeptin‐miR‐105–1 axis.

## Introduction

The control of reproductive processes involves the intricate integration of various factors, encompassing intracellular regulators such as microRNAs (miRNAs) and extracellular regulators like the neuropeptide kisspeptin.

MiRNAs are small (19–25 nucleotides), noncoding RNA molecules that regulate gene expression by binding to complementary sequences in the 3′ untranslated region of target mRNAs, thus leading to their inhibition of translation, degradation, or both [1]. Substantive evidence supporting the involvement of miRNAs in the regulation of ovarian functions has emerged from the characterization of miRNA profiles in normal human ovarian cells [2], dynamic alterations during ovarian development [3], and malignant transformations [4]. Direct insight into the regulatory actions of several miRNAs in granulosa cells, spanning cellular growth, development, proliferation, apoptosis, and steroid hormone synthesis and release, has been garnered through studies involving transfection-based miRNA overexpression or silencing [3, 5–7].

MiR-105 has been suggested to be involved in the control of human ovarian functions. MiR-105 has been identified as a potent tumor suppressor with significant implications in the progression of ovarian cancer, as evidenced by studies conducted by Lam et al. [8] and Li et al. [9]. Notably, its involvement in the apoptosis of ovarian cancer cells has been elucidated by Kou et al. [10]. In healthy human ovarian granulosa cells, miR-105 has been implicated in promoting proliferation while concurrently diminishing apoptosis and the production of progesterone, testosterone, and estradiol [11, 12]. However, the role of miR-105–1 in the control of porcine ovarian cell functions has not yet been studied.

Further potential mechanism of miRNAs influence on ovarian processes could be their cooperation with upstream hormonal regulator kisspeptin. The presence of kisspeptin and the expression of kisspeptin receptor mRNA in human and animal ovarian granulosa cells, including those of porcine origin, have been reported in various studies [13–16]. Recent in vitro studies have demonstrated the ability of kisspeptin to alter various facets of ovarian cell physiology, including viability, proliferation, apoptosis, and the release of steroid and peptide hormones, as well as prostaglandin E2, across different mammalian species, including pigs [15–19]. These data suggest a potential direct role of kisspeptin in the regulation of both human and animal ovarian functions, along with steroidogenesis, follicullogenesis and luteogenesis.

Moreover, the previous study indicated that the effect of kisspeptin on bovine ovarian progesterone synthesis could be mediated by miR-1246 [19]. Nevertheless, the functional interrelationships between miR-105–1 and kisspeptin in the control of ovarian cell functions remain to be studied.

For this purpose, we examined the following mechanisms: (a) the effect of miR‐105–1 regulators and kisspeptin on basic porcine ovarian granulosa cell functions (viability, proliferation, apoptosis, progesterone, estradiol, and IGF‐I release); (b) the ability of porcine granulosa cells to produce kisspeptin; (c) the modulatory potential of miR-105–1 on the accumulation and actions of kisspeptin in ovarian cells; and (d) the potential of kisspeptin to affect the expression and effects of miR‐105–1.

## Materials and Methods

### Oligonucleotides

MiR-105–1 mimics (double-stranded RNAs that mimic mature endogenous miR-105–1 and enhance miRNA activity, representing a gain-of-function assay), miR-105–1 inhibitor (single-stranded 2-O-methyl-modified oligonucleotide fragments that reduce endogenous miRNA activity, representing a loss-of-function assay), and their respective negative controls (NC) (Table 1) were purchased from GenePharma Co., Ltd. (Shanghai, China). Synthesized and purified through high-performance liquid chromatography, these oligonucleotides exhibited a purity exceeding 97%, validated by mass spectrometry.

**Table 1:** The sequences of miRNA mimics and miRNA inhibitor

Oligonucleotides	Sequence	
miR-105–1 mimics	Sense	5′-UCAAAUGCUCAGACUCCUGU-3′
	Antisense	5′-AGGAGUCUGAGCAUUUGAUU-3′
miR-NC (negative control) mimics	Sense	5′-UUCUCCGAACGUGUCACGUTT-3′
	Antisense	5′-ACGUGACACGUUCGGAGAATT-3′
miR-105–1 inhibitor	Sense	5′-ACAGGAGUCUGAGCAUUUGA-3′
miR-NC inhibitor	Sense	5′-CAGUACUUUUGUGUAGUACAA-3′

### Preparation, Transfection, and Culture of Ovarian Granulosa Cells

Twenty porcine ovaries were collected from Landrace prepubertal gilts (6–8 months of age) at the slaughterhouse of Chovmat F.U. in Rastislavice (Slovakia). The ovaries were individually preserved in a thermos containing a physiological solution and processed within 6 h of slaughter.

Ovarian granulosa cells were isolated from porcine ovarian (4.5–6.5 mm diameter) follicles without visible signs of atresia (including weak vascularization, thin follicular walls, and pale follicular fluid) by using aspiration with a syringe. Following aspiration and cell isolation via centrifugation for 10 min at 1.500 rpm, the granulosa cells were rinsed in sterile DMEM/F12 1:1 medium (cat. no. 31331093; Thermo Fisher Scientific, Waltham, MA, USA) and resuspended in the same medium with 10% fetal calf serum (cat. no. 092910154; MP Biomedicals, Santa Ana, California, USA) and 1% antibiotic–antimycotic solution (cat. no. 091674049; MP Biomedicals). Cells were counted by using a Buerker chamber, and their concentration was adjusted to the required volume (10^6^ cells/ml medium). The cell suspension was dispensed in 24-well culture plates (cat. no. 142475; Thermo Fisher Scientific; 1 ml suspension/well) for ELISA procedures, 96-well culture plates (cat. no. 781962; Brand®, Wertheim, Germany; 200 μl/well) for XTT (sodium 3′-[1- (phenylaminocarbonyl)- 3,4-tetrazolium]-bis (4-methoxy6-nitro) benzene sulfonic acid hydrate), TUNEL (Terminal deoxynucleotidyl transferase dUTP Nick-End Labeling), and BrdU assays, or 16-well chamber slides (cat. no. 16260681; Thermo Fisher Scientific; 200 μl/well) for immunocytochemistry. Preculturing the cells at 37.5 °C in 5% CO_2_ allowed the formation of a 75% confluent monolayer within 2–3 days.

### Experimental Setup

In the first series of experiments, investigations focused on the effects of miR-105–1 mimics and inhibitor on miR-105–1 levels, basic ovarian functions, and kisspeptin occurrence. Transfection of ovarian granulosa cells with miR-105–1 mimics, miR-105–1 inhibitor, and their corresponding negative controls was facilitated using Lipofectamine® RNAiMAX Transfection Reagent (cat. no. 13778150; Thermo Fisher Scientific), according to the manufacturer's protocol. A final oligonucleotide concentration of 25 nM was employed, with nontransfected and negative control-transfected cells serving as control groups.

In the second series of experiments studying the effects of miR-105–1 mimics, kisspeptin, and their combinations on basic ovarian functions, after 24 h of transfection, granulosa cells were cultured with and without kisspeptin-10 (0, 1, and 10 ng/ml; cat. no. AS-64240; Eurogentec, Seraing, Belgium). These doses of the shortest biologically active kisspeptin were comparable with the effective doses used in previous similar in vitro experiments [15–17]. Control groups included nontransfected or transfected cells without kisspeptin treatment.

After culture, the culture medium and cells were processed for immunocytochemistry, XTT, TUNEL, and BrdU assays, RT‒qPCR, and enzyme immunoassay (ELISA). Furthermore, cell concentration and viability were determined by using the Trypan blue exclusion test and a hemocytometer.

### Cell Viability Test

The Trypan blue exclusion test (0.4%) assessed cell viability as per established protocols [20, 21]. Briefly, the medium was removed from the culture plates after incubation of the granulosa cells. Subsequently, the cell monolayer was subjected to Trypan blue staining (cat. no. 091691049; MP Biomedicals) for 15 min. After Trypan blue treatment, cells were fixed for 30 min in 4% paraformaldehyde. After fixation, plates were washed with a physiological solution and subjected to microscopic inspection (magnification: 400 ×). The ratio of dead (stained) cells to the total cell count was calculated.

### XTT Assay

The cell proliferative activity was evaluated by using the XTT Cell Proliferation Assay Kit (cat. no. ab232856; Abcam, Cambridge, UK) according to the manufacturer's instructions. XTT is a yellow tetrazolium salt that is reduced to an orange formazan product by mitochondrial reductases in metabolically active proliferating cells [22]. Briefly, the activated-XTT solution was added to each well and incubated for two hours at 37 °C and 5% CO2. Absorbance (abs) was read at 450 nm by using an ELISA reader (cat. no. BS-050108-A02; Biosan, Riga, Latvia). The percentage of proliferative active cells was calculated by using the following equation:

$$\%\;\mathrm o\mathrm f\;\mathrm X\mathrm T\mathrm T\;\mathrm f\mathrm o\mathrm r\mathrm m\mathrm a\mathrm z\mathrm a\mathrm n-\mathrm{positive}\;\mathrm{cell}=\frac{Abs\;\left(treated\;cells\right)-Abs\;(blank)}{Abs\;\left(control\;cells\right)-Abs\;(blank)}\times100$$

### BrdU Assay

Cell proliferation, based on the measurement of 5-bromo-2′-deoxyuridine (BrdU) incorporation during DNA synthesis, was determined by using colorimetric cell proliferation ELISA (cat. no. 11647229001; Sigma-Aldrich, Saint-Louis, MO, USA) according to the manufacturer's instructions. The reaction products were quantified by measuring the absorbance at 450 nm using an ELISA reader (cat. no. BS-050108-A02; Biosan).

### TUNEL Assay

DNA fragmentation induced in the cell culture was measured by using the TUNEL assay (HT TiterTACS™ Apoptosis Detection Kit; cat. no. 4822–96-K; R&D Systems, Minneapolis, MN, USA) following the manufacturer's instructions. The absorbance was measured at 450 nm by using an ELISA reader (cat. no. BS-050108-A02; Biosan) after adding 0.2 N HCl. Negative controls involved cells labeled without transferase terminal deoxynucleotidyl transferase (TdT), while positive controls were generated by using TACS-Nuclease for one hour at 37 °C before hydrogen peroxide treatment.

### Immunocytochemical Analysis of the Presence of Kisspeptin, Proliferation, and Apoptosis Markers

The presence of the kisspeptin protein, markers of proliferation (PCNA and cyclin B1), and apoptosis (bcl-2, bax, caspase 3, and p53) were detected via immunocytochemistry [23]. After washing and fixation, the cells were incubated in a blocking solution with 1% bovine serum albumin (cat. no. 9048–46-8; Sigma-Aldrich) at room temperature for 1 h, to prevent non-specific binding of the antiserum. Next, the cells were incubated with primary antibodies listed in Table 2 for 1 h at room temperature. To detect the binding sites of the primary antibody, the cells were incubated with secondary antibody for 1 h at room temperature listed in Table 2. To visualize positive signals, the cells labelled with horseradish peroxidase were stained with 3,3′-diaminobenzidine (DAB) substrate (cat. no. 11718096001; Sigma-Aldrich) for 1 h. Following DAB-staining, the cells on chamber slides were washed with PBS and covered with a drop of Glycergel mounting medium (cat. no. C056330-2; Agilent Technologies, Santa Clara, CA, USA). A coverslip was then attached to the microslide, and the cells were visualized using a light microscope. Cells labelled with CFL 594 were mounted in VECTASHIELD Antifade Mounting Medium with 4′,6-diamidino-2-phenylindole (DAPI), a selective stain for cell nuclear DNA (cat. no. H-1200–10; Vector Laboratories, Inc., Burlingame, CA, USA). DAPI and CFL 594-labeled secondary antibodies were detected using fluorescence microscopy. Cells treated without the primary antibody were used as negative controls. The number of stained cells and the location of intracellular molecules were determined based on the brown coloration of DAB peroxidase or the red fluorescence emitted by the CFL 594 label using a light or fluorescence microscope (Leica Microsystems, Wetzlar, Germany) and the IM500 Leica software. The ratio of stained cells to the total number of cells was determined.

**Table 2:** Antibodies, source, and commercially purchased reagents used

Antibodies	Company	Catalog number	pAB/mAB*	Dilution
Primary antibodies				
KiSS-1	Santa Cruz Biotechnology	sc-101246	mAB	1:500
PCNA	Santa Cruz Biotechnology	sc-25280	mAB	1:500
cyclin B1	Santa Cruz Biotechnology	sc-245	mAB	1:500
bcl 2	Proteintech	26,593–1-AP	pAB	1:200
bax	Santa Cruz Biotechnology	sc-23959	mAB	1:500
caspase 3	Santa Cruz Biotechnology	sc-7272	mAB	1:500
p53	Santa Cruz Biotechnology	sc-393031	mAB	1:500
Secundary antibodies				
Anti-mouse IgG-HRP	Santa Cruz Biotechnology	sc-516102	pAB	1:100
Anti-mouse IgG-CFL 594	Santa Cruz Biotechnology	sc-516178	pAB	1:500

^*^pAB, polyclonal antibody; mAB, monoclonal antibody

### RT‒qPCR for miR-105–1

After treatment with miR-105–1 mimics and miR-105–1 inhibitor for 48 h, total RNA from transfected granulosa cells was extracted by using TRIzol Reagent (Invitrogen) according to the manufacturer's instructions. The concentration and quality of total RNA were measured by a Quantus Fluorometer (cat. no. E6150; Promega, Madison, WI, USA). Mature miR-105–1 expression levels were quantified utilizing the Hairpin-it miRNAs qPCR kit (cat. no. QPM-010; GenePharma Co., Ltd.) on an ABI 7500 Fast Instrument (cat. no. 4351104; Thermo Fisher Scientific). The amplification thermocycling conditions were as follows: an initial denaturation at 95 °C for 3 min, followed by 40 cycles at 95 °C for 10 s, annealing and elongation at 60 °C for 10 s, and 60 °C for 60 s. U6 small nuclear RNA (snRNA) was used as an internal control, and relative gene expression was calculated by using the 2^–ΔΔCt^ method [24]. The sequences of the utilized primers (Table 3) were designed and synthesized by GenePharma Co., Ltd. All of the samples were analyzed in triplicate from the same RNA preparation, and the mean values were calculated.

**Table 3:** The sequences of gene primers for RT-qPCR

Gene symbol	Primer sequence	
miR-105–1	Forward	5′-TGGTTCGCTCAAATGCTCAG-3′
	Reverse	5′-TATGGTTGTTCACGACTCCTTCAC-3
U6	Forward	5′-CTCGCTTCGGCAGCACA-3′
	Reverse	5′-AACGCT TCACGAATTTGCGT-3′

### Enzyme-Linked Immunoassay (ELISA)

The concentrations of progesterone (cat. no. FR E-2500), *17β*-estradiol (cat. no. FR E-2000), and IGF-1 (cat. no. ME E-0500) were determined in 25 µl aliquots of the incubation medium by using an ELISA according to the manufacturer's instructions (LDN Immunoassays and Services, Nodhorn, Germany). The characteristics of these assays are presented in Table 4. This ELISA was validated for the culture medium samples by using dilution tests.

**Table 4:** Characteristics of the immunoassays used in experiments

Substance assayed	Specificity of assay (cross-reactivity of antiserum)	Sensitivity of assay (ng/ml)	Coefficient of variation (%)	
			Intra-assay	Inter-assay
Progesterone	≤ 1.1% with 11-desoxycorticosterone, ≤ 0.35% with pregnenolone, ≤ 0.30% 17α-OH with progesterone, ≤ 0.20% with corticosterone, < 0.10% with estriol, 17β-estradiol, testosterone, cortisone and 11-desoxycortisol, < 0.02% with DHEA-S and cortisol	0.045	5.4	5.59
*17β*-estradiol	≤ 9.5% with fulvestrant, ≤ 4.2% with estrone, ≤ 3.8% with E2-3-glucuronide, ≤ 3.6% with E2-3-sulphate, ≤ 0.4% with estriol, < 0.1% with androstenedione, 17-hydroxyprogesterone, corticosterone, pregnenolone, E2-17-glucuronide, progesterone, and testosterone	0.0062	6.4	4.5
IGF-I	100% with IGF-I, ≤ 3.3% with insulin, and 1.02% with IGF-II	9.75	7.39	12.63

### Statistical Analysis

The data from this study are reported as the means of values that were obtained in three separate experiments performed on separate days with different groups of granulosa cells, each obtained from at least six ovaries. Each experimental group was represented by four culture wells containing ovarian granulosa cells. For the Trypan blue exclusion test, the rates of viability were calculated from at least 100 cells per well. For the immunocytochemical analysis, the proportion of cells containing antigens was calculated from at least 1,000 cells per well. For the ELISA, blank control values were subtracted from corresponding values that were determined for media-containing cells to exclude any nonspecific background (less than 10% of the total values). The rates of substance secretion were calculated per 10^6^ viable cells/day. Significant differences between the groups were determined by using the Shapiro‒Wilk normality and Student's t tests, as well as one-way ANOVA followed by Tukey's tests, with SigmaPlot 11.0 (Systat Software, GmbH, Erkrath, Germany). Differences were compared for statistical significance at P levels less than 0.05 (*P* < 0.05).

## Results

### Transfection Efficiency

**Fig. 1:**
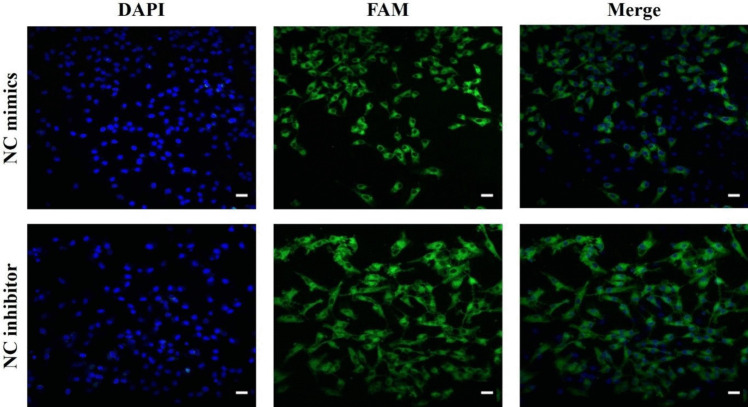
Transfection efficiency of miR-105-1 mimics and inhibitor in porcine granulosa cells after 48 h transfection with these oligonucleotides. Fluorescence microscopy analysis of green fluorescence-labeled (FAM-labeled) negative control (NC) mimics and FAM-labeled NC inhibitor. DAPI was used to stain the cellular nuclei. Scale bars: 1 cm = 20 μm

**Fig. 2:**
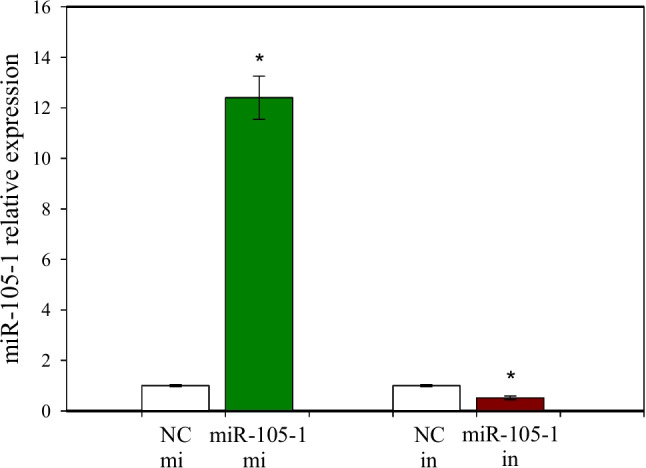
Evaluation of miR-105-1 expression levels by reverse transcription-quantitative polymerase chain reaction (RT‒qPCR) in cells transfected with miR-105-1 mimics (mi), miR-105-1 inhibitor (in), or their respective negative controls (NC). Values are the means ± SEMs. Significance vs. control: *, *P *< 0.05

In the present study, porcine ovarian granulosa cells underwent transfection with miR-105–1 mimics, miR-105–1 inhibitor, or their respective negative controls (NC). The transfection efficiency exceeded 81%, as confirmed by the transfection of fluorescein-labeled (FAM-labeled) NC mimics and NC inhibitor (Fig. 1). Further validation through RT‒qPCR revealed a significant increase in miR-105–1 expression in granulosa cells following miR-105–1 mimics transfection, while the opposite effect was observed with the miR-105–1 inhibitor (Fig. 2).

### Effects of miR-105–1 on Viability, Proliferation, Apoptosis, and Secretory Activity

**Fig. 3:**
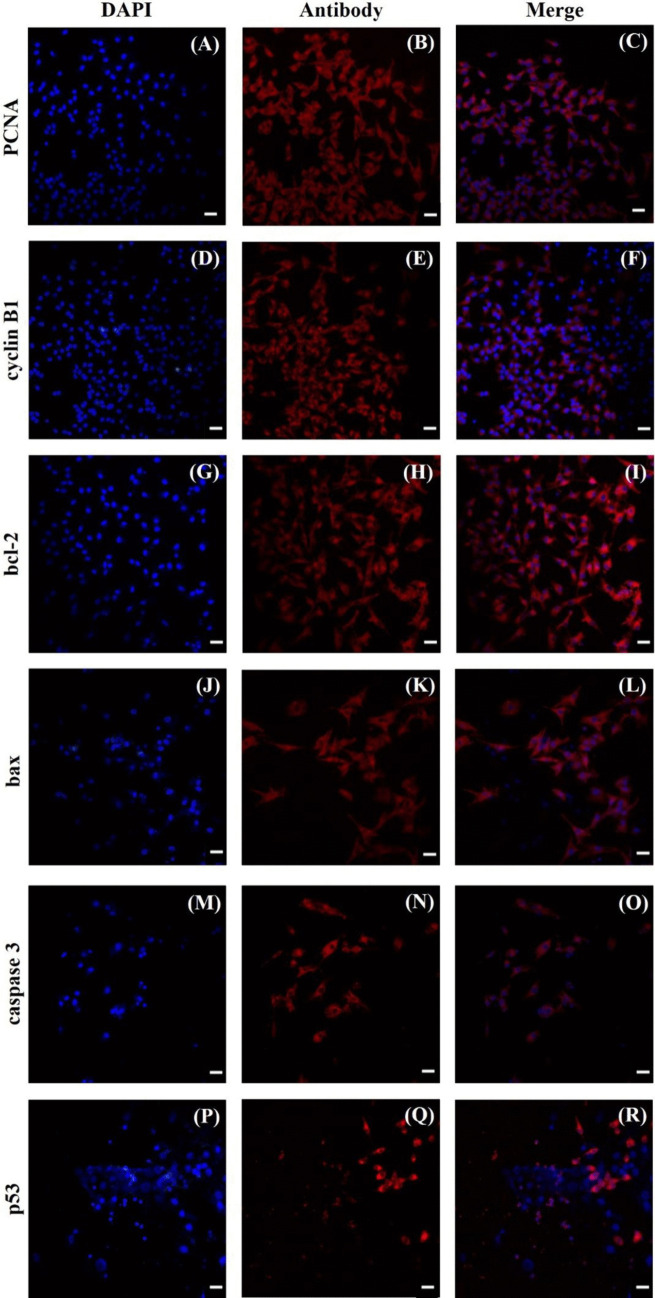
Fluorescent immunostaining of PCNA, cyclin B1, bcl-2, bax, caspase 3, and p53 in cultured porcine ovarian granulosa cells. Specific staining was performed by using primary antiserum against PCNA (**B**), cyclin B1 (**E**), bcl-2 (**H**), bax (**K**), caspase 3 (**N**), and p53 (**Q**) and secondary antiserum labeled with CruzFluor™ 594 (CFL 594, red fluorescence). Then, 4′,6-diamidino-2-phenylindole (DAPI) was used to stain the cellular nuclei (**A**, **D**, **G**, **J**, **M**, **P**) (blue fluorescence). (**C**) is the merged image of (**A**) and (**B**). (**F**) is the merged image of (**D**) and (**E**). (**I**) is the merged image of (**G**) and (**H**). (**L**) is the merged image of (**J**) and (**K**). (**O**) is the merged image of (**M**) and (**N**). (**R**) is the merged image of (**P**) and (**Q**). Scale bars: 1 cm = 20 μm

**Fig. 4:**
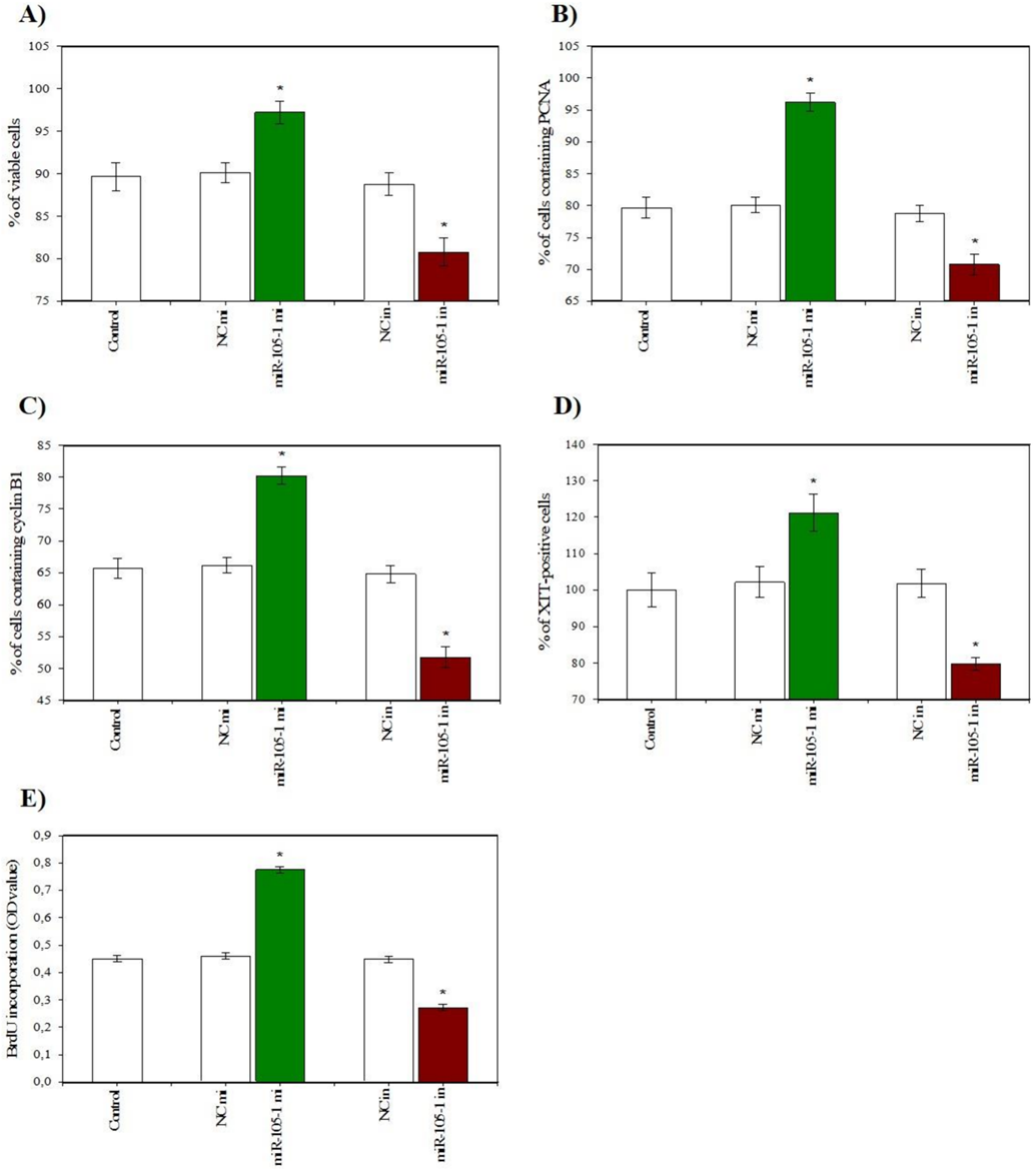
Effect of miR-105-1 mimics (mi) and miR-105-1 inhibitor (in) on cell viability (**A**), proliferation (accumulation of PCNA (**B**), cyclin B1 (**C**), XTT- (**D**), and BrdU-positive cells (**E**)) in cultured porcine ovarian granulosa cells. The results show (*) the effects of miR-105-1 mi or miR-105-1 in, with a significant (*P *< 0.05) difference between the cells transfected with negative control mimics (NC mi) or negative control inhibitor (NC in) and with miR-105-1 mi or miR-105-1 in. The results are expressed as the mean ± SEM from at least 3 independent experiments

**Fig. 5:**
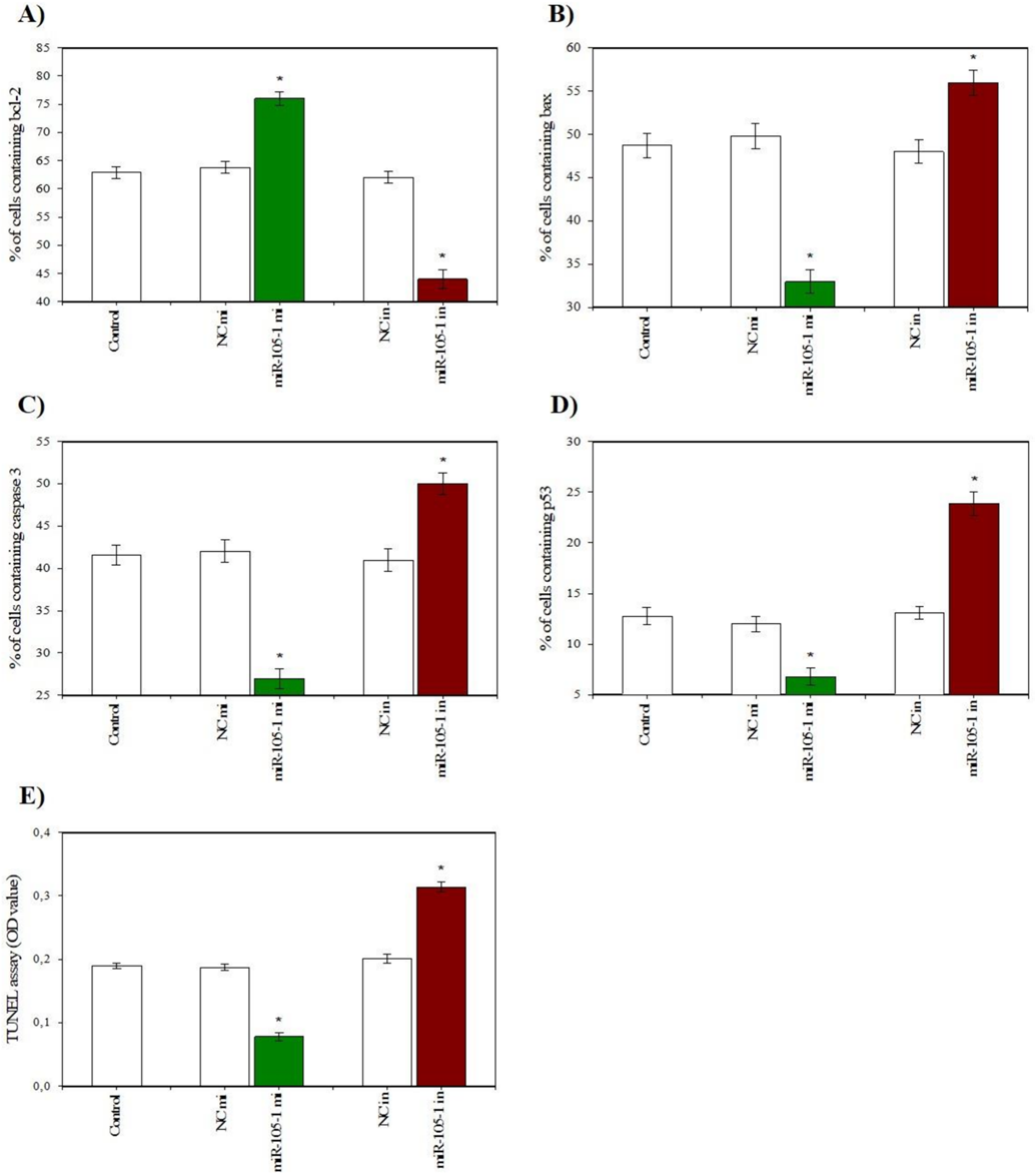
Effect of miR-105-1 mimics (mi) and miR-105-1 inhibitor (in) on cell apoptosis (accumulation of bcl-2 (**A**), bax (**B**), caspase 3 (**C**), p53 (**D**), and TUNEL-positive cells (**E**)) in cultured porcine ovarian granulosa cells. The results show (*) the effects of miR-105-1 mi or miR-105-1 in, with a significant (*P* < 0.05) difference between the cells transfected with negative control mimics (NC mi) or negative control inhibitor (NC in) and with miR-105-1 mi or miR-105-1 in. The results are expressed as the mean ± SEM from at least 3 independent experiments

**Fig. 6:**
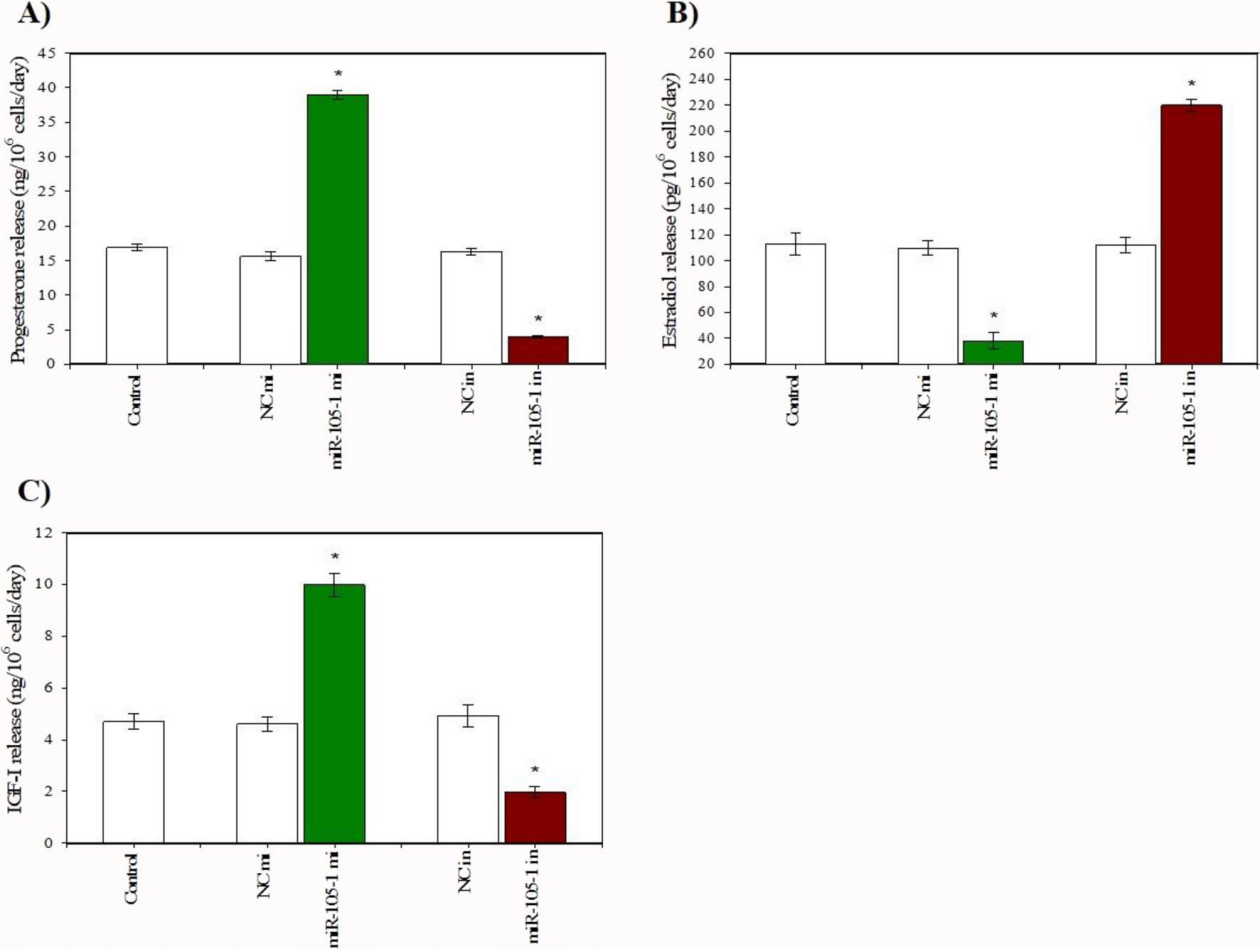
Effect of miR-105-1 mimics (mi) and miR-105-1 inhibitor (in) on the release of progesterone (**A**), estradiol (**B**), and IGF-I (**C**) in cultured porcine ovarian granulosa cells. The results show (*) the effects of miR-105-1 mi or miR-105-1 in, with a significant (*P* < 0.05) difference between the cells transfected with negative control mimics (NC mi) or negative control inhibitor (NC in) and with miR-105-1 mi or miR-105-1 in. The results are expressed as the mean ± SEM from at least 3 independent experiments

The cells that were collected after culture contained visible staining for PCNA, cyclin B1, bcl-2, bax, caspase 3, and p53. Cyclin B1 was predominantly localized in cell nuclei, while PCNA, bcl-2, bax, caspase 3, and p53 were predominantly present in the cytoplasm (Fig. 3). Transfection with miR-105–1 mimics enhanced cell viability, the percentage of proliferation-related peptides (PCNA and cyclin B), and the proportion of actively proliferating cells (XTT formazan- and BrdU-positive cells) (Fig. 4A, B, C, D, E). Additionally, miR-105–1 mimics increased the proportion of antiapoptotic peptide bcl-2 (Fig. 5A), while reducing the accumulation of proapoptotic proteins (bax, caspase 3, and p53) and DNA fragmented cells (TUNEL-positive cells) (Fig. 5B, C, D, E). Furthermore, miR-105–1 mimics increased the progesterone and IGF-I output but inhibited estradiol release (Fig. 6A, B, C).

Conversely, the miR-105–1 inhibitor exhibited an inhibitory effect on cell viability, proliferation-related proteins (PCNA and cyclin B1), and the XTT formazan- and BrdU-positive cells (Fig. 4A, B, C, D, E). . The miR-105–1 inhibitor also reduced the proportion of the antiapoptotic protein bcl-2 and promoted the accumulation of the proapoptotic peptides (bax, caspase 3, and p53) and TUNEL-positive cells (Fig. 5A, B, C, D, E). . Additionally, the miR-105–1 inhibitor suppressed progesterone and IGF-I release, while stimulating estradiol output (Fig. 6A, B, C).

### Effects of miR-105–1 on the Occurrence Of Kisspeptin

**Fig. 7:**
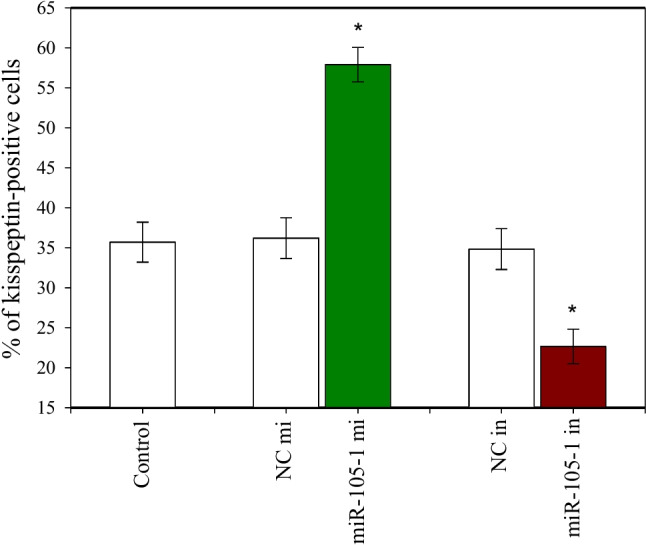
Effect of miR‐105-1 mimics (mi) and miR‐105-1 inhibitor (in) on the proportion of porcine ovarian granulosa cells containing kisspeptin. After 2 days of culture, the cells were analyzed by immunocytochemistry. The results show (*) the effects of miR‐101-5 mi or miR-105-1 in, with a significant (*P* < 0.05) difference between the cells transfected with negative control mimics (NC mi) or negative control inhibitor (NC in) and with miR‐105-1 mi or miR‐105-1 in. The results are expressed as the mean ± SEM from at least 3 independent experiments

The percentage of cells containing kisspeptin varied between 22.65% and 57.89%, and this percentage was affected by transfection of cells with miR‐105–1 mimics and miR‐105–1 inhibitor. MiR‐105–1 mimics increased and miR-105–1 inhibitor decreased the accumulation of kisspeptin in the cells (Fig. 7).

### Effects of Kisspeptin on miR-105–1 Expression, Viability, Proliferation, Apoptosis, and Secretory Activity

**Fig. 8:**
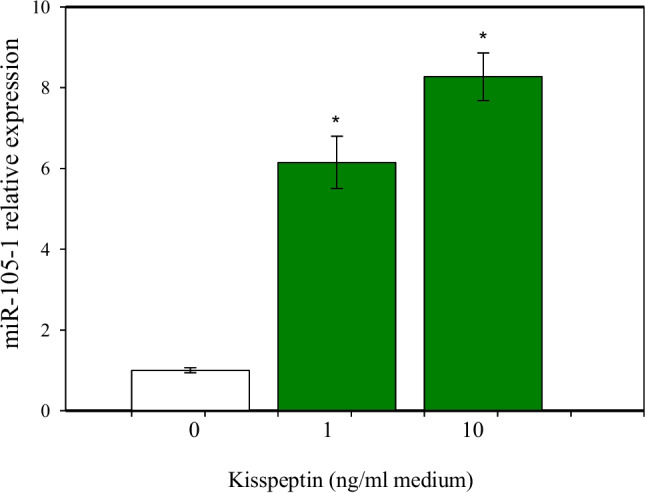
Effect of the administration of kisspeptin (0, 1, and 10 ng/ml) on miR-105-1 expression levels in porcine ovarian granulosa cells. The results show (*) the effect of kisspeptin, with a significant (*P* < 0.05) difference between the cells cultured without (0 ng/ml) and with kisspeptin (1 and 10 ng/ml). The results are expressed as the mean ± SEM from at least 3 independent experiments

**Fig. 9:**
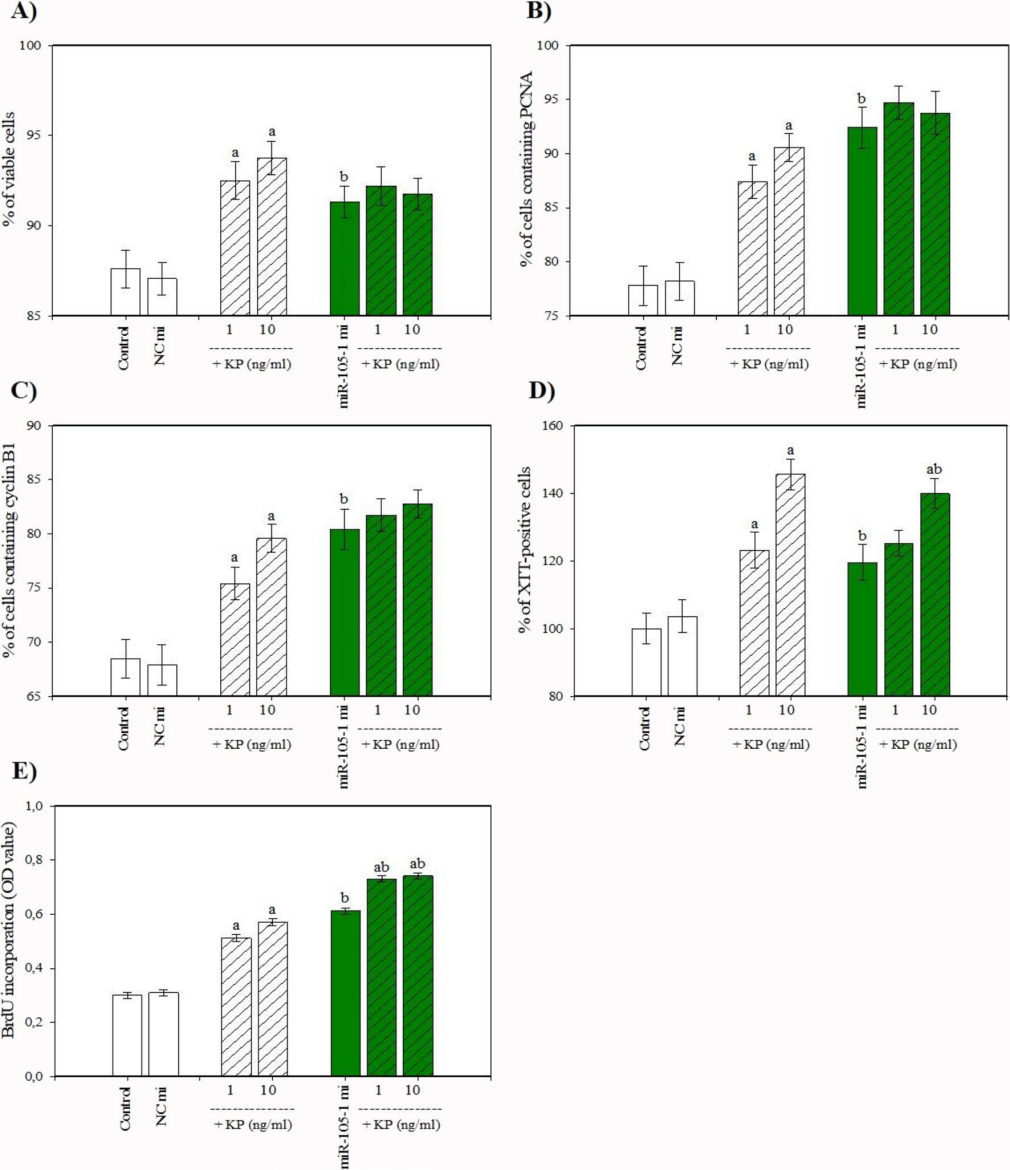
Effect of administration of kisspeptin (KP) (0, 1, and 10 ng/ml) alone and in combination with miR-105-1 mimics (mi) on cell viability (**A**) and proliferation (accumulation of PCNA (**B**), cyclin B1 (**C**), XTT- (**D**), and BrdU-positive cells (**E**)) in cultured porcine ovarian granulosa cells. The results show (a) significant (P<0.05) differences between the cells treated without (0 ng/ml) and with KP alone; (b) significant (P<0.05) differences between the cells treated without and with miR-105-1 mi alone; and (ab) significant (*P* < 0.05) differences between the cells treated with KP alone and KP in combination with miR-105-1 mi. The results are expressed as the mean ± SEM from at least 3 independent experiments

**Fig. 10:**
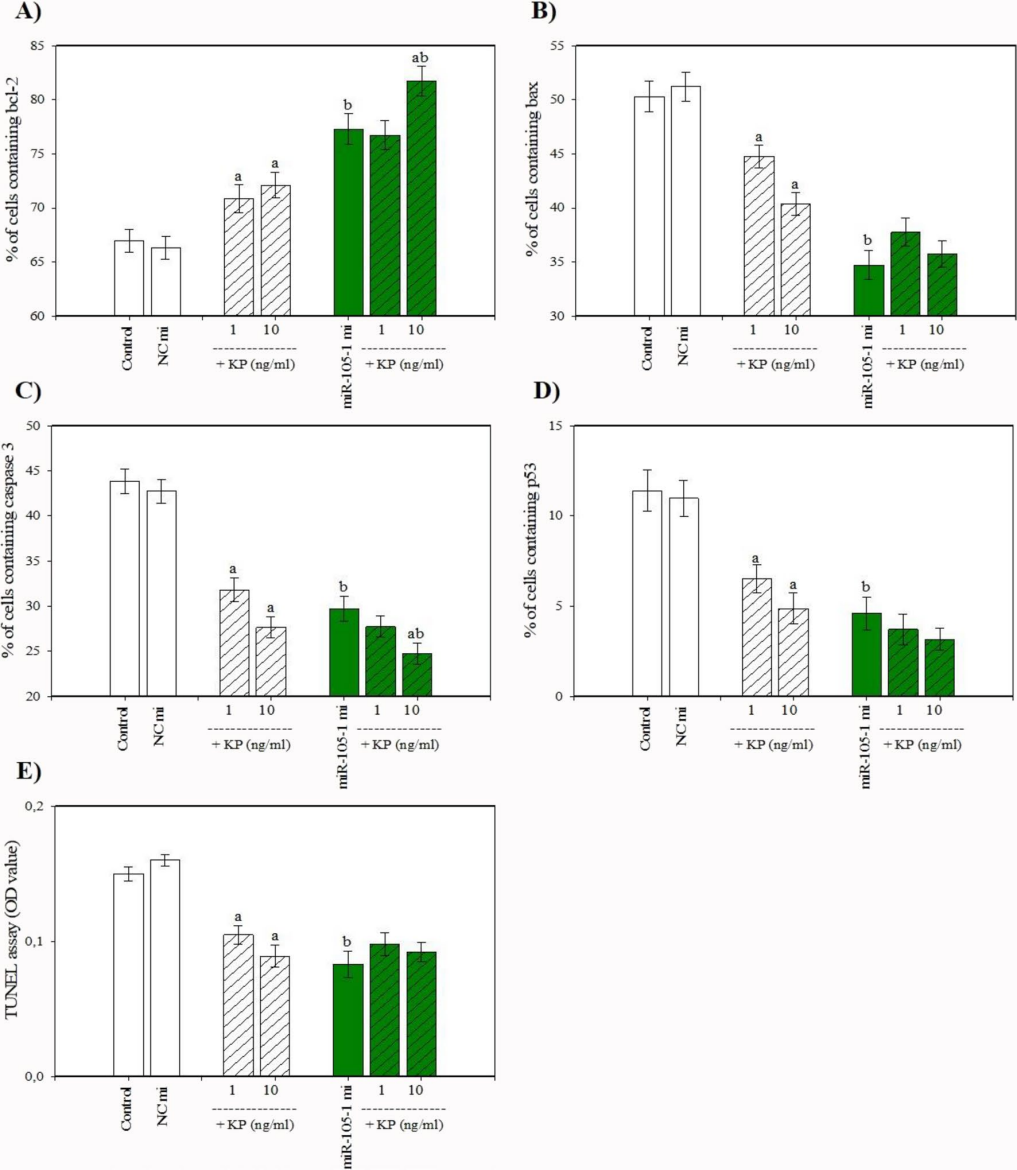
Effect of administration of kisspeptin (KP) (0, 1, and 10 ng/ml) alone and in combination with miR-105-1 mimics (mi) on cell apoptosis (accumulation of bcl-2 (**A**), bax (**B**), caspase 3 (**C**), p53 (**D**), and TUNEL-positive cells (**E**)) in cultured porcine ovarian granulosa cells. The results show (a) significant (*P* < 0.05) differences between the cells treated without (0 ng/ml) and with KP alone; (b) significant (P<0.05) differences between the cells treated without and with miR-105-1 mi alone; and (ab) significant (P<0.05) differences between the cells treated with KP alone and KP in combination with miR-105-1 mi. The results are expressed as the mean ± SEM from at least 3 independent experiments

**Fig. 11:**
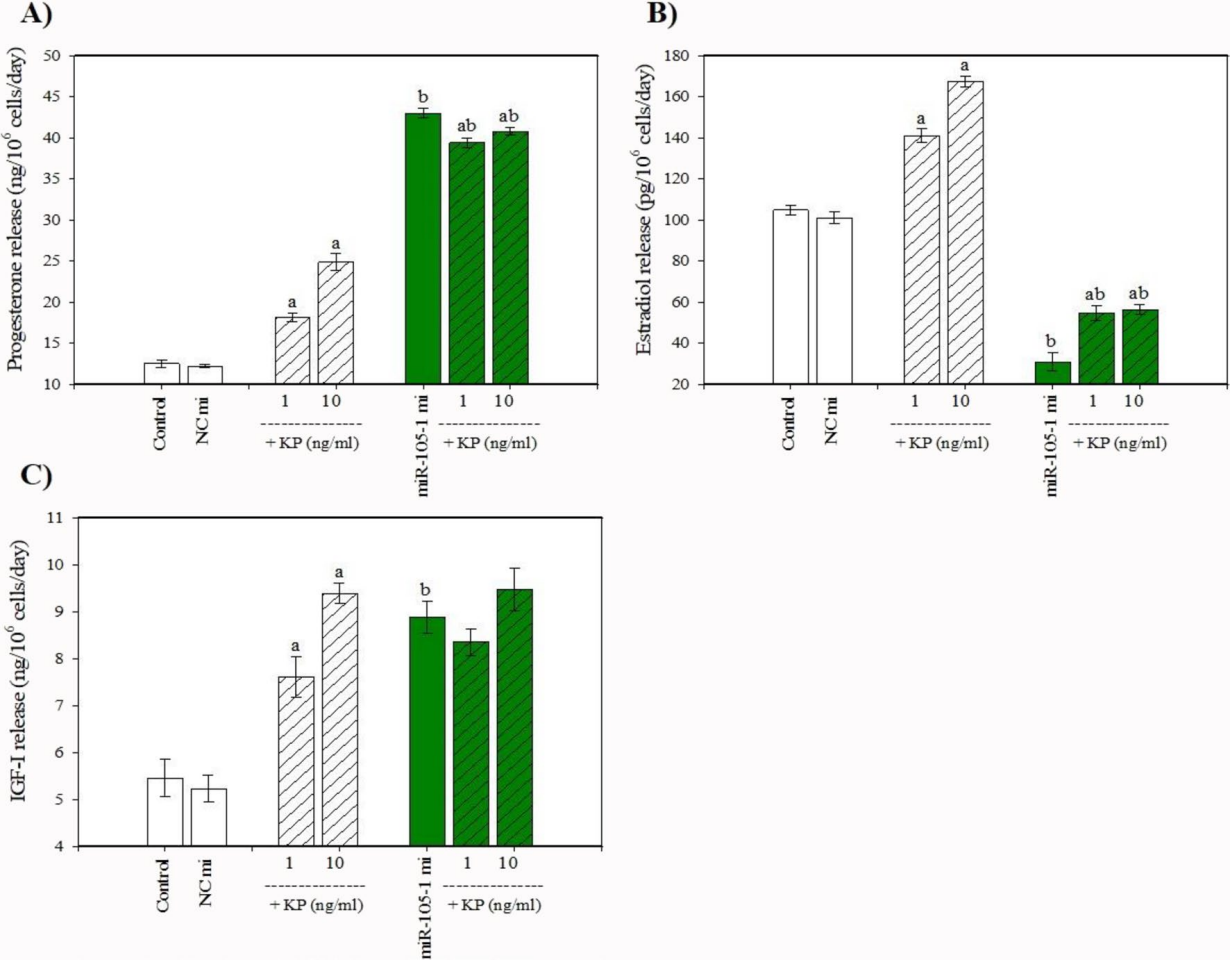
Effect of administration of kisspeptin (KP) (0, 1, and 10 ng/ml) alone and in combination with miR-105-1 mimics (mi) on the release of progesterone (**A**), estradiol (**B**), and IGF-I (**C**) in cultured porcine ovarian granulosa cells. The results show (a) significant (*P* < 0.05) differences between the cells treated without (0 ng/ml) and with KP alone; (b) significant (*P* < 0.05) differences between the cells treated without and with miR-105-1 mi alone; and (ab) significant (P<0.05) differences between the cells treated with KP alone and KP in combination with miR-105-1 mi. The results are expressed as the mean ± SEM from at least 3 independent experiments

When administered alone, kisspeptin stimulated miR-105–1 expression levels (Fig. 8), cell viability, and the accumulation of PCNA-, cyclin B1-, XTT formazan-, and BrdU-positive cells (Fig. 9A, B, C, D, E). Kisspeptin also increased the proportion of bcl-2-positive cells and inhibited apoptotic cells (bax-, caspase 3-, p53-, and TUNEL-positive) (Fig. 10A, B, C, D, E). Moreover, kisspeptin promoted progesterone, estradiol, and IGF-I release (Fig. 11A, B, C).

### Effects of miR-105–1 in Combination with Kisspeptin on Viability, Proliferation, Apoptosis, and Secretory Activity

Transfecting cells with miR-105–1 mimics enhanced the effect of kisspeptin on the accumulation of XTT formazan-, bcl-2-, and caspase 3- (at a dose of 10 ng/ml; Figs. 9D, 10A, C) and BrdU-positive cells, and the release of progesterone (at doses of 1 and 10 ng/ml; Figs. 9E, 11A). It also reversed its impact on estradiol output (at doses of 1 and 10 ng/ml; Fig. 11B) but did not alter its effects on cell viability, PCNA-, cyclin B1-, bax-, p53-, and TUNEL-positive cells or IGF-I release (Figs. 9A, B, C, 10B, D, E, 11C). Kisspeptin, in turn, supported the stimulatory actions of miR-105–1 mimics on PCNA-, cyclin B1-, and BrdU-positive cells, bcl-2-positive cell accumulation, and progesterone release (at doses of 1 and 10 ng/ml; Figs. 9B, C, E, 10A, 11A) and its inhibitory effect on the percentage of bax-, caspase 3-, and p53-positive cells and estradiol output (at doses of 1 and 10 ng/ml; Figs. 10B, C, D, 11B) but did not modify its influence on cell viability and the accumulation of XTT formazan- and TUNEL-positive cells (Figs. 9A, D, 10E).

## Discussion

The presence of kisspeptin, proliferation- and apoptosis-related molecules and the abilities of cells to exclude Trypan blue, to secrete steroid hormones and IGF-I by cultured ovarian cells, as well as the ability of both control and transfected cells to respond to kisspeptin, indicated that these cells were viable and suitable for the present study. The results of fluorescence microscopy and RT‒qPCR of transfection efficiency and the expression of miR-105–1 confirm previous data on the efficiency and reproducibility of the miRNA delivery method that was used [3].

Proliferation is a crucial process in ovarian physiology, which plays a significant role in folliculogenesis and oocyte growth [25]. The orchestrated modifications in morphological and physiological attributes of granulosa cells during follicle formation are intricately associated with cellular proliferation. Our experimental findings revealed that when porcine granulosa cells were transfected with miR-105–1 mimics, there was a notable increase in the accumulation of proliferation-related proteins, such as PCNA and cyclin B1, as well as an increase in the proportion of proliferative active cells, as determined by XTT formazan and BrdU positivity. However, we observed an inhibitory effect on cell proliferation when we transfected the cells with an miR-105–1 inhibitor. Our results provide the first evidence of the pro-proliferative effect of miR-105–1 on porcine ovarian cells, aligning with the findings of Sirotkin et al. [12], who delineated a similar stimulatory effect of miR-105–1 on human granulosa cell proliferation, substantiated through the expression of PCNA and cyclin B1. The action of miR-105–1 on the proportion of PCNA (endogenous promoter and marker of the S-phase of mitosis) [26], BrdU (exogenous marker of DNA synthesis) [27], and cyclin B1 (governing the transition from G2 to M phase of the cell cycle) [28], implies that miR-105–1 exerts a stimulatory effect on mitosis at G2 and S phases through the modulation of cyclin B1, PCNA, and BrdU. In addition, these observations suggest that miR-105–1 can increase cell proliferation, probably through downregulating its inhibitor, p53 [29].

Previous studies have reported that miR-105–1 promotes apoptosis in human ovarian carcinoma cells [10]. Our research has demonstrated that increased cell proliferation in the ovary may be linked to decreased cell apoptosis, an essential process involved in oogenesis, folliculogenesis, oocyte selection, and atresia [30]. We found that administration of miR-105–1 mimics stimulated the accumulation of the the antiapoptotic marker bcl-2 and inhibited the proportion of cytoplasmic/mitochondrial/intrinsic apoptosis (bax, caspase 3) [31, 32] in porcine granulosa cells. In the intrinsic apoptotic pathway, antiapoptotic protein Bcl-2 resides in the outer mitochondrial wall and prevents cytochrome c release. The proapoptotic bax allows cytochrome c to leak out of the mitochondria in response to death signals [33]. The analysis of the present results regarding the molecules that regulate mitochondrial functions showed that miR-105–1 decreased the proportion of bax. Moreover, previous studies have demonstrated that bcl-2 and bax are present in the granulosa cells of both fetal and adult ovaries, suggesting their possible role in atresia. Bcl-2 has been observed in growing follicles, whereas Bax has been observed in atretic follicles [34]. Caspases are primary effector molecules involved in ovarian apoptosis. In the ovary, caspase-3 is expressed in luteal and theca cells of healthy corpora lutea, as well as in granulosa cells of atretic follicles [33]. The current study revealed that that miR-105–1 mimics exhibit a capability to diminish nuclear/extrinsic apoptosis, typified by the suppression of p53 [35, 36] and DNA fragmentation (TUNEL-positive cells) [37]. According to recent studies, the expression of p53 protein in apoptotic ovarian cells from atretic follicles might indicate its involvement in atresia. Moreover, inhibition of p53 expression has been proven to results in a substantial decrease in the number of apoptotic ovarian cells and atretic follicles [36]. Contrastingly, the miR-105–1 inhibitor demonstrated an opposing effect on these apoptosis-related proteins. It's worth noting that this study is the first to investigate the anti-apoptotic role of miR-105–1 on porcine granulosa cells. Our findings are consistent with earlier reports by Sirotkin et al. [12] that suggest that miR-105–1 can inhibit apoptosis in human ovarian cells.

In the present experiments, miR-105–1 mimics promoted cell viability; moreover, miR-105–1 inhibitor had the opposite effect. The orchestration of cell viability and ovarian folliculogenesis is intricately intertwined with the proliferation/apoptosis rate of ovarian cells [38]. The increase in this rate can explain the miR-105–1-induced promotion of cell viability. Therefore, the possibility that miR-105–1 can suppress ovarian follicular atresia and support ovarian folliculogenesis (thus promoting fecundity) should not be excluded. Furthermore, miR-105–1 can promote ovarian cell viability through changes in the release of ovarian steroid and peptide hormones – established regulators of cellular viability, proliferation, apoptosis, and ovarian follicular development or atresia [25].

The results of the present study demonstrate that miR-105–1 mimics stimulate progesterone and IGF-I and inhibit estradiol release from porcine ovarian granulosa cells. In contrast, the miR-105–1 inhibitor had an inhibitory effect on the output of progesterone and IGF-I; furthermore, it increased estradiol release. These observations are consistent with the study by Sirotkin et al. [11], who reported that miR-105 reduced the output of estradiol release by human granulosa cells. In addition, this is the first evidence for miR-105–1 involvement in the secretory activity of porcine ovarian cells, discerned through the release of steroid hormones and IGF-I.

The ability of miR-105–1 to affect the release of steroid hormones and IGF-I may indicate its involvement in the control of some hormone-dependent ovarian processes. For example, progesterone emerges as a marker and promoter of ovarian cell luteinization, while estradiol and IGF-I act as stimulators of follicle recruitment, growth, and development [25]. Therefore, our data indicate that miR-105–1 may be considered a potential regulator of ovarian follicle survival, growth, and follicular luteinization.

The complexity of the interrelationships between different steroid hormones and IGF-I, as affected by miR-105–1, requires further examination. The influence of miR-105–1 on some hormones could be a primary effect (although other effects may be secondary) through changes in hormone precursors. For example, progesterone serves as a precursor to testosterone, and the aromatization of testosterone results in estradiol production [39]. Therefore, changes in estradiol release may be due to changes in its precursor production. Additionally, estradiol can exert negative feedback mechanisms to curtail the excessive production of progesterone. Moreover, the influence of miR-105–1 on ovarian steroid release may extend through IGF-I, a potent regulator of ovarian steroidogenesis [40].

**Fig. 12:**
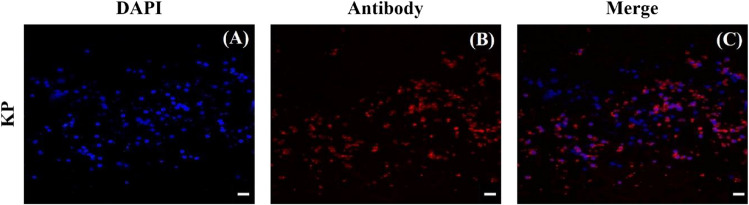
Fluorescent immunostaining of kisspeptin (KP) in cultured porcine ovarian granulosa cells. (**B**) Specific staining by using primary antiserum against KP and secondary antiserum labeled with fluorescein CruzFluor™ 594 (CFL 594; red fluorescence). DAPI was used to stain the cellular nuclei (**A**) (blue fluorescence). (**C**) is the merged image of (**A**) and (**B**). Scale bars: 1 cm = 20 μm

Immunocytochemical analysis showed the presence of kisspeptin in porcine ovarian granulosa cells after culture. Kisspeptin immunoreactivity was detected in both the nucleus and cytoplasm of granulosa cells (Fig. 12). These findings substantiate earlier reports by Basini et al. [14] regarding the presence of kisspeptin in cultured porcine ovarian granulosa cells.

In the present investigation, kisspeptin treatment stimulated cell viability and proliferation and the release of progesterone, estradiol, and IGF-I and inhibited apoptosis. These observations confirm the previous data concerning the putative role of this neuropeptide in the regulation of ovarian functions [14–19]. These observations may indicate kisspeptin involvement in supporting ovarian follicle recruitment and their further development, which are promoted by ovarian cell proliferation and suppression of their apoptosis under the influence of estradiol and IGF-I. In addition, kisspeptin could be involved in the promotion of ovarian cell luteinization, which is characterized by an increase in ovarian cell proliferation and the output of progesterone [25].

Our data are the first to demonstrate the existence of a functional interrelationship between kisspeptin and miR-105–1 in the ovary. In the present experiments, kisspeptin treatment increased miR-105–1 levels in granulosa cells. This observation suggests that kisspeptin exerts a regulatory influence on miR-105–1 expression, thereby potentially modulating miR-105–1-dependent processes within the ovary. Moreover, kisspeptin emerges as both a regulator and a promoter of the effects of miR-105–1, supporting the stimulatory influence of miR-105–1 mimics on cell proliferation and progesterone release, while inhibiting apoptosis and estradiol release.

On the other hand, the current study demonstrated that miR-105–1 might also be a modifier of kisspeptin action. MiR‐105–1 can modify the effects of kisspeptin on ovarian cells via changes in the percentage of kisspeptin-positive cells. At least, transfection of cells with miR‐105–1 mimics upregulated and miR-105–1 inhibitor downregulated the proportion of kisspeptin in porcine granulosa cells. Moreover, miR‐105–1 mimics supported the action of kisspeptin on proliferation, apoptosis, and progesterone and reversed its influence on estradiol release. This finding may indicate that miR-105–1 can affect ovarian processes via changes in kisspeptin, kisspeptin receptors, or kisspeptin postreceptor signaling pathways, as described in several publications [41–43].

The understanding of the mechanism and biological importance of kisspeptin-miRNA interrelationships requires further studies. Nevertheless, the ability of kisspeptin to promote miR-105–1 expression and the ability of miR-105–1 to increase the occurrence of kisspeptin and modify kisspeptin effects indicate the existence of a self-stimulating kisspeptin-miR-105–1 axis, with miR-105–1 potentially serving as a novel mediator of kisspeptin action on the ovary.

However, a key limitation of this study was that it was an in vitro study. Thus, the results of the current study need to be verified and explained through additional in vitro and in vivo animal and clinical studies to convert the current hypotheses regarding the nature, mechanisms, and practical applications of the miR-105–1-kisspeptin axis into rational mechanistic theories and technologies for animal production, assisted reproduction, and reproductive medicine. Furthermore, future research should: a) explore the signaling pathways and gene targets of miR-105–1, their physiological roles, and their potential applications; b) investigate the connections between miR-105–1 and kisspeptin and the molecules that regulate them; c) identify the specific expression patterns of miR-105–1 under certain ovarian physiological and pathological conditions, which could be used as additional diagnostic tools to evaluate these disorders; d) develop reliable methods of measuring this miRNA for use as quantitative marker to characterize and predict the state of certain tissues and cells; and e) gain insights into novel treatments for some ovarian disorders by combining miR-105–1 or kisspeptin and traditional pharmaceutical drugs.

## Conclusion

The current data elucidate the first evidence for the role of miR‐105–1 in the control of basic porcine ovarian cell processes (viability, proliferation, apoptosis, the release of steroid hormones and IGF-I) and their response to kisspeptin effects.

From a practical viewpoint, the results suggest the potential usefulness of miR-105–1 regulators for the control of ovarian follicular growth and development, as well as in the promotion and synchronization of the cell cycle. In addition, these observations indicate the potential ability of miR-105–1 to suppress both cytoplasmic and nuclear apoptosis via the downregulation of their promoters; therefore, they may be able to inhibit apoptosis-related ovarian events. For example, miR-105–1 mimics may suppress ovarian follicular atresia (due to a decrease in ovarian cell apoptosis), thus promoting ovarian folliculogenesis and fecundity [25]. Furthermore, we cannot exclude the possibility that miR-105–1 may be applicable as a diagnostic and predictive marker for some disorders and for the development of novel therapies for hormone-related syndromes and tumorigenesis, which require the suppression of ovarian cell functions.

Moreover, the stimulatory action of kisspeptin on ovarian cells points toward a promising avenue for enhancing reproductive outcomes in both animals and humans. This encompasses the facilitation of ovarian hormone release, ovarian folliculogenesis, and luteogenesis, as well as the potential promotion and synchronization of estrous cycles. Successful applications of kisspeptin in inducing puberty and ovulation in farm animals [44] and humans [45] further accentuate its reproductive prowess.

Finally, the ability of miR-105–1 to promote the occurrence and action of kisspeptin indicates the next area of its application. Co-administration of miR-105–1 with kisspeptin holds promise in elevating the efficacy of kisspeptin-based treatments for ovulation induction in assisted reproduction, animal production, and the management of ovarian insufficiency. We cannot exclude the possibility that the combination of miR-105–1 with kisspeptin could have better stimulatory and therapeutic effects than the effect of these regulators given alone. All these hypotheses, however, require further validation by in vitro and in vivo studies.

## Data Availability

The authors confirm that the data supporting the findings of this study are available within the article and its supplementary materials.

## Declarations

### Animal Welfare Statement

The authors confirm that the ethical policies of the journal, as noted on the journal’s author guidelines page, have been adhered to. No ethical approval was required as the present studies did not use living animals, that they did not require animal killing or other manipulations affecting animal welfare.

## References

[1] O'Brien J, Hayder H, Zayed Y, Peng C. Overview of microRNA biogenesis, mechanisms of actions, and circulation. Front Endocrinol (Lausanne). 2018;9:402. 10.3389/fendo.2018.00402 10.3389/fendo.2018.00402 PMC6085463 30123182

[2] Salas-Huetos A, James ER, Aston KI, Jenkins TG, Carrell DT, Yeste M. The Expression of miRNAs in Human Ovaries, Oocytes, Extracellular Vesicles, and Early Embryos: A Systematic Review. Cells. 2019;8(12):1564. 10.3390/cells8121564. 31817143 10.3390/cells8121564 PMC6952888

[3] Tu J, Cheung AH, Chan CL, Chan WY. The Role of microRNAs in Ovarian Granulosa Cells in Health and Disease. Front Endocrinol (Lausanne). 2019;10:174. 10.3389/fendo.2019.00174. 30949134 10.3389/fendo.2019.00174 PMC6437095

[4] Alshamrani AA. Roles of microRNAs in ovarian cancer tumorigenesis: two decades later, what have we learned? Front Oncol. 2020;10:1084. 10.3389/fonc.2020.01084 10.3389/fonc.2020.01084 PMC7396563 32850313

[5] Toms D, Pan B, Li J. Endocrine regulation in the ovary by microRNA during the estrous cycle. Front Endocrinol (Lausanne). 2018;8:378. 10.3389/fendo.2017.00378 10.3389/fendo.2017.00378 PMC5786742 29403434

[6] Reza AMMT, Choi YJ, Han SG, et al. Roles of microRNAs in mammalian reproduction: from the commitment of germ cells to peri-implantation embryos. Biol Rev Camb Philos Soc. 2019;94(2):415–38. 10.1111/brv.12459. 30151880 10.1111/brv.12459 PMC7379200

[7] Azhar S, Dong D, Shen WJ, Hu Z, Kraemer FB. The role of miRNAs in regulating adrenal and gonadal steroidogenesis. J Mol Endocrinol. 2020;64(1):R21–43. 10.1530/JME-19-0105. 31671401 10.1530/JME-19-0105 PMC7202133

[8] Lam SS, Ip CK, Mak AS, Wong AS. A novel p70 S6 kinase-microRNA biogenesis axis mediates multicellular spheroid formation in ovarian cancer progression. Oncotarget. 2016;7(25):38064–77. 10.18632/oncotarget.9345. 27191261 10.18632/oncotarget.9345 PMC5122372

[9] Li M, Zhang S, Ma Y, Yang Y, An R. Role of hsa-miR-105 during the pathogenesis of paclitaxel resistance and its clinical implication in ovarian cancer. Oncol Rep. 2021;45(5):84. 10.3892/or.2021.8035. 33846814 10.3892/or.2021.8035 PMC8025119

[10] Kou X, Ding H, Li L, Chao H. Hsa-miR-105–1 regulates cisplatin-resistance in ovarian carcinoma cells by targeting ANXA9. Anal Cell Pathol (Amst). 2021;2021:6662486. 10.1155/2021/6662486 10.1155/2021/6662486 PMC7929659 33680718

[11] Sirotkin AV, Ovcharenko D, Grossmann R, Lauková M, Mlyncek M. Identification of microRNAs controlling human ovarian cell steroidogenesis via a genome-scale screen. J Cell Physiol. 2009;219(2):415–20. 10.1002/jcp.21689. 19194990 10.1002/jcp.21689

[12] Sirotkin AV, Lauková M, Ovcharenko D, Brenaut P, Mlyncek M. Identification of microRNAs controlling human ovarian cell proliferation and apoptosis. J Cell Physiol. 2010;223(1):49–56. 10.1002/jcp.21999. 20039279 10.1002/jcp.21999

[13] García-Ortega J, Pinto FM, Fernández-Sánchez M, et al. Expression of neurokinin B/NK3 receptor and kisspeptin/KISS1 receptor in human granulosa cells. Hum Reprod. 2014;29(12):2736–46. 10.1093/humrep/deu247. 25316443 10.1093/humrep/deu247

[14] Basini G, Grasselli F, Bussolati S, et al. Presence and function of kisspeptin/KISS1R system in swine ovarian follicles [published correction appears in Theriogenology. 2019 Aug;134:141-142]. Theriogenology. 2018;115:1-8. 10.1016/j.theriogenology.2018.04.006 10.1016/j.theriogenology.2019.05.082 31203854

[15] Fabová Z, Loncová B, Mlyn Ek M, Sirotkin AV. Interrelationships between amphiregulin, kisspeptin, FSH and FSH receptor in promotion of human ovarian cell functions. Reprod Fertil Dev. 2022;34(3):362–77. 10.1071/RD21230. 35109967 10.1071/RD21230

[16] Fabová Z, Loncová B, Mlynček M, Sirotkin AV. Kisspeptin as autocrine/paracrine regulator of human ovarian cell functions: Possible interrelationships with FSH and its receptor. Reprod Biol. 2022;22(1):100580. 10.1016/j.repbio.2021.100580. 34844165 10.1016/j.repbio.2021.100580

[17] Fabová Z, Sirotkin AV. Interrelationships between kisspeptin and FSH in control of porcine ovarian cell functions. Domest Anim Endocrinol. 2021;74:106520. 10.1016/j.domaniend.2020.106520. 32738561 10.1016/j.domaniend.2020.106520

[18] Dai T, Kang X, Yang C, et al. Integrative analysis of miRNA-mRNA in ovarian granulosa cells treated with kisspeptin in tan sheep. Animals (Basel). 2022;12(21):2989. 10.3390/ani12212989 10.3390/ani12212989 PMC9656243 36359113

[19] Guo L, Xu H, Li Y, et al. Kisspeptin-10 promotes progesterone synthesis in bovine ovarian granulosa cells via downregulation of microRNA-1246. Genes (Basel). 2022;13(2):298. Published 2022 Feb 3. 10.3390/genes13020298 10.3390/genes13020298 PMC8871966 35205342

[20] Perry SW, Epstein LG, Gelbard HA. In situ trypan blue staining of monolayer cell cultures for permanent fixation and mounting. Biotechniques. 1997;22(6):1020–4. 10.2144/97226bm01. 9187742 10.2144/97226bm01

[21] Uzuner SÇ. Development of a direct trypan blue exclusion method to detect cell viability of adherent cells into ELISA plates. Celal Bayar Universitesi Fen Bilimleri Dergisi. 2018;14:99–104. 10.18466/cbayarfbe.372192

[22] Berridge MV, Herst PM, Tan AS. Tetrazolium dyes as tools in cell biology: new insights into their cellular reduction. Biotechnol Annu Rev. 2005;11:127–52. 10.1016/S1387-2656(05)11004-7. 16216776 10.1016/S1387-2656(05)11004-7

[23] Osborn M, Brandfass S. Immunocytochemistry of frozen and of paraffin tissue sections. In: Celis JE, editor. Cell biology: a laboratory handbook, 3rd edn. Amsterdam: Elsevier; 2006. pp. 563–569.

[24] Livak KJ, Schmittgen TD. Analysis of relative gene expression data using real-time quantitative PCR and the 2(-Delta Delta C(T)) Method. Methods. 2001;25(4):402–8. 10.1006/meth.2001.1262. 11846609 10.1006/meth.2001.1262

[25] Sirotkin AV. Regulators of ovarian functions. Nova Science Pub; 2014.

[26] Shiomi Y, Nishitani H. Control of genome integrity by RFC complexes; conductors of PCNA loading onto and unloading from chromatin during DNA replication. Genes (Basel). 2017;8(2):52. 10.3390/genes8020052 10.3390/genes8020052 PMC5333041 28134787

[27] Martí-Clúa J. Incorporation of 5-Bromo-2'-deoxyuridine into DNA and proliferative behavior of cerebellar neuroblasts: all that glitters is not gold. Cells. 2021;10(6):1453. 10.3390/cells10061453 10.3390/cells10061453 PMC8229392 34200598

[28] Nakayama Y, Yamaguchi N. Role of cyclin B1 levels in DNA damage and DNA damage-induced senescence. Int Rev Cell Mol Biol. 2013;305:303–37. 10.1016/B978-0-12-407695-2.00007-X. 23890385 10.1016/B978-0-12-407695-2.00007-X

[29] Mollereau B, Ma D. The p53 control of apoptosis and proliferation: lessons from Drosophila. Apoptosis. 2014; 19(10):1421–1429. 10.1007/s10495-014-1035-7 10.1007/s10495-014-1035-7 PMC4167030 25217223

[30] Kulus J, Kulus M, Kranc W, Jopek K, Zdun M, Józkowiak M, Jaśkowski JM, Piotrowska Kempisty H, Bukowska D, Antosik P, et al. Transcriptomic Profile of New Gene Markers Encoding Proteins Responsible for Structure of Porcine Ovarian Granulosa Cells. Biology. 2021;10(11):1214. 10.3390/biology10111214. 34827207 10.3390/biology10111214 PMC8615192

[31] An LS, Yuan XH, Hu Y, et al. Progesterone production requires activation of caspase-3 in preovulatory granulosa cells in a serum starvation model. Steroids. 2012;77(13):1477–82. 10.1016/j.steroids.2012.07.011. 22963862 10.1016/j.steroids.2012.07.011

[32] Peña-Blanco A, García-Sáez AJ. Bax, Bak and beyond - mitochondrial performance in apoptosis. FEBS J. 2018;285(3):416–31. 10.1111/febs.14186. 28755482 10.1111/febs.14186

[33] Ola MS, Nawaz M, Ahsan H. Role of Bcl-2 family proteins and caspases in the regulation of apoptosis. Mol Cell Biochem. 2011;351(1–2):41–58. 10.1007/s11010-010-0709-x. 21210296 10.1007/s11010-010-0709-x

[34] Albamonte MI, Albamonte MS, Bou-Khair RM, Zuccardi L, Vitullo AD. The ovarian germinal reserve and apoptosis-related proteins in the infant and adolescent human ovary. J Ovarian Res. 2019;12(1):22. 10.1186/s13048-019-0496-2. 30857552 10.1186/s13048-019-0496-2 PMC6410530

[35] Ranjan A, Iwakuma T. Non-canonical cell death induced by p53. Int J Mol Sci. 2016;17(12):2068. 10.3390/ijms17122068 10.3390/ijms17122068 PMC5187868 27941671

[36] Napoli M, Flores ER. The p53 family orchestrates the regulation of metabolism: physiological regulation and implications for cancer therapy. Br J Cancer. 2017;116:149–55. 10.1038/bjc.2016.384. 27884017 10.1038/bjc.2016.384 PMC5243983

[37] Mirzayans R, Murray D. Do TUNEL and other apoptosis assays detect cell death in preclinical studies? Int J Mol Sci. 2020;21(23):9090. 10.3390/ijms21239090 10.3390/ijms21239090 PMC7730366 33260475

[38] Holesh JE, Bass AN, Lord M. Physiology, ovulation. Treasure Island, FL. 2023 28723025

[39] Das N, Kumar TR. Molecular regulation of follicle-stimulating hormone synthesis, secretion and action. J Mol Endocrinol. 2018;60(3):R131–55. 10.1530/JME-17-0308. 29437880 10.1530/JME-17-0308 PMC5851872

[40] Skowronski MT, Mlotkowska P, Tanski D, et al. Pituitary gonadotropins, prolactin and growth hormone differentially regulate AQP1 expression in the porcine ovarian follicular cells. Int J Mol Sci. 2017;19(1):5. 10.3390/ijms19010005 10.3390/ijms19010005 PMC5795957 29267208

[41] Hu KL, Zhao H, Chang HM, Yu Y, Qiao J. Kisspeptin/kisspeptin receptor system in the ovary. Front Endocrinol (Lausanne). 2018;8:365. Published 2018 Jan 4. 10.3389/fendo.2017.00365 10.3389/fendo.2017.00365 PMC5758547 29354093

[42] Cao Y, Li Z, Jiang W, Ling Y, Kuang H. Reproductive functions of kisspeptin/KISS1R systems in the periphery. Reprod Biol Endocrinol. 2019;17(1):65. Published 2019 Aug 9. 10.1186/s12958-019-0511-x 10.1186/s12958-019-0511-x PMC6689161 31399145

[43] D’Occhio MJ, Campanile G, Baruselli PS. Peripheral action of kisspeptin at reproductive tissues-role in ovarian function and embryo implantation and relevance to assisted reproductive technology in livestock: a review. Biol Reprod. 2020;103(6):1157–70. 10.1093/biolre/ioaa135. 32776148 10.1093/biolre/ioaa135 PMC7711897

[44] Caraty A, Decourt C, Briant C, Beltramo M. Kisspeptins and the reproductive axis: potential applications to manage reproduction in farm animals. Domest Anim Endocrinol. 2012;43(2):95–102. 10.1016/j.domaniend.2012.03.002. 22533939 10.1016/j.domaniend.2012.03.002

[45] Szeliga A, Podfigurna A, Bala G, Meczekalski B. Kisspeptin and neurokinin B analogs use in gynecological endocrinology: where do we stand? J Endocrinol Invest. 2020;43(5):555–61. 10.1007/s40618-019-01160-0. 31838714 10.1007/s40618-019-01160-0

